# The implications of circadian rhythms for anaesthesia

**DOI:** 10.1016/j.bjae.2026.06.005

**Published:** 2026-07-23

**Authors:** G.R. Warman, R.L. de Souza, D. Cumin, J.F. Cheeseman

**Affiliations:** 1 University of Auckland, Auckland, New Zealand; 2 Auckland Hospital, Health New Zealand, New Zealand

**Keywords:** anaesthesia, chronobiology, circadian rhythms, sleep

**Key points**

• The circadian clock has an influence on the action of many drugs used in anaesthesia.
• This time-of-day influence on drug action can alter duration of analgesia and time to recovery.
• Conversely, many drugs used for anaesthesia disrupt patients’ circadian clock and sleep; this may contribute to postoperative delirium.
• Giving melatonin before and after surgery, using α_2_-agonists such as dexmedetomidine, and strengthening the daily light cycle in hospital may help to improve patients’ sleep and recovery.

**Learning objectives**

By reading this article, you should be able to:

• Detail the effect of time of day on drug action and how this may explain some of the interpatient variability in drug responses
• Explain how and why anaesthesia can disrupt patients’ circadian clocks
• Describe the usefulness of melatonin and light in supporting circadian rhythms
• Consider how these effects may influence the practice of anaesthesia

Although anaesthetists often regard humans as essentially homeostatic, our physiology and behaviour are in fact characterised by robust, predictable 24-h oscillations generated by the circadian clock (Fig. 1).^1^ These rhythms influence nearly every major biological system and, consequently, produce substantial time-of-day variation in both the pharmacokinetics and pharmacodynamics of many drugs.^2^ Despite long-standing recognition that circadian biology has important implications for anaesthesia, its relevance is frequently underappreciated in clinical settings.^2^ The duration of anaesthesia produced by a standard drug dose varies systematically with the time of day it is given, and recent work has demonstrated that this relationship is bidirectional, with anaesthetic agents themselves affecting the clock, producing measurable shifts in circadian phase and subsequent physiological and behavioural rhythms.^3^^,^^4^ It is increasingly clear that anaesthesia interacts with the circadian system in ways that extend well beyond the immediate perioperative period and that, by considering circadian effects on drug action and the effects of anaesthetic drugs on the clock, there is the potential to better explain interpatient variability in the response to drugs and even to improve postoperative patient sleep and expedite time to discharge.^5^

**Fig 1:**
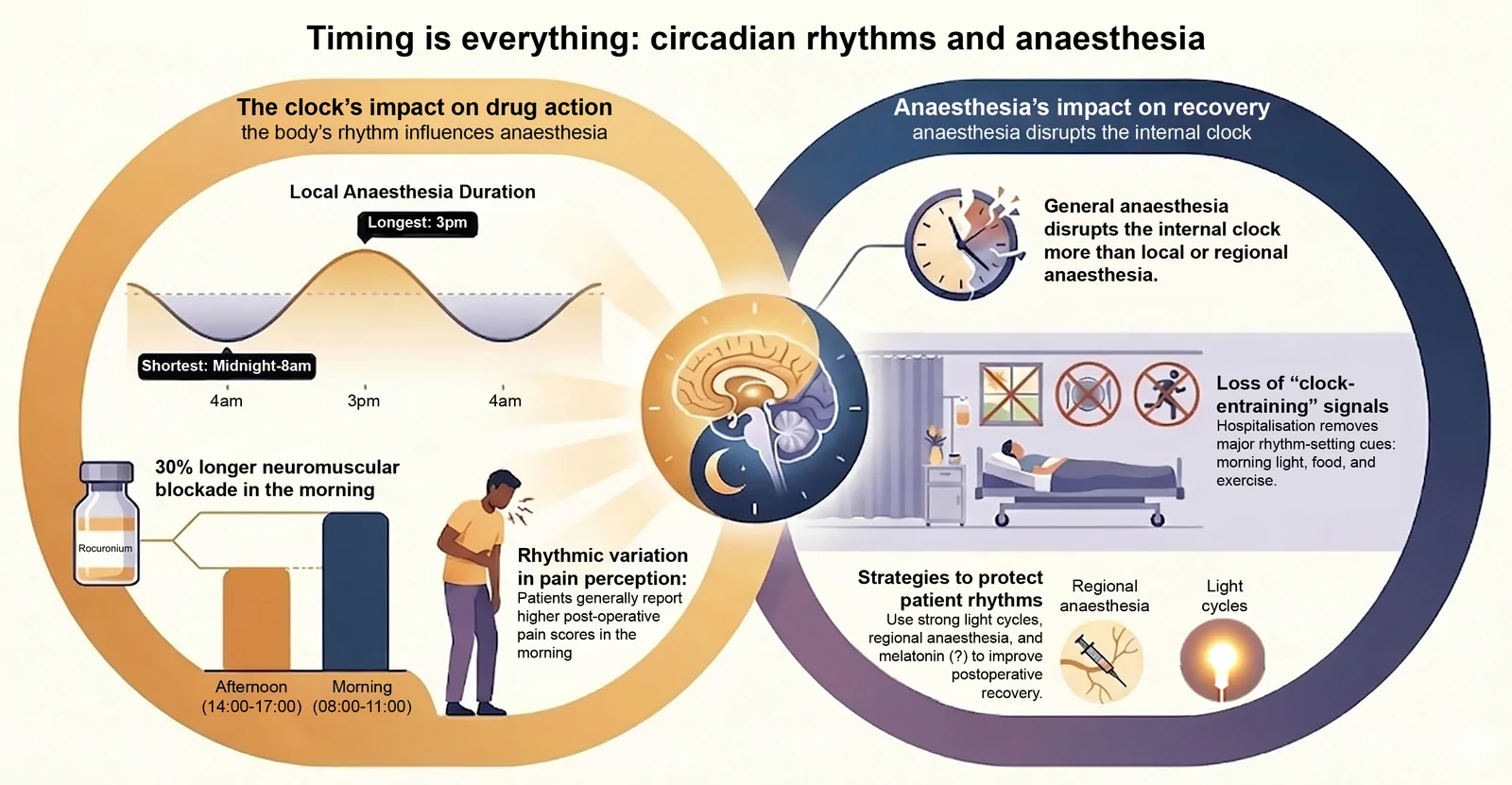
Summary of the impact the circadian clock and time of day have on anaesthetic drug action (yellow) and the corollary effects of anaesthesia on the circadian clock (blue).

In this review, we first describe the effect the circadian clock has on the action of anaesthetic drugs. We then turn our attention to the influence that anaesthesia (and surgery) has on the clock itself and discuss its implications for postoperative patient recovery. We conclude by highlighting how this may influence anaesthetic practice.

## The circadian clock

The daily rhythms of animals and plants have been noted for thousands of years, and the proof that these rhythms are generated by the action of an endogenous circadian clock was first demonstrated in the 1700s. However, it was the discovery in the 1970s and 1980s of the genes that drive the circadian clock that led to a rapid expansion of the research area known as *chronobiology—*the study of biological rhythms and the clocks that control them.^1^

We now know that circadian clocks exist in all organisms, from unicellular bacteria to humans, and that they drive daily rhythms in everything from gene expression to behaviour.^6^ These clocks affect all aspects of our functioning as humans, from appetite to immunity, from sleepiness to pain, and from cognitive function to drug absorption.^7^, ^8^, ^9^ The presence of robust circadian rhythms is increasingly understood to be essential for health and wellbeing.^6^ Furthermore, disruptions to our rhythms can even provide early indicators of disease and mortality.^10^

The central circadian clock of mammals resides in the paired suprachiasmatic nuclei (SCN) of the hypothalamus, a group of some 50,000 cells containing a set of clock genes (the so-called clock genes *period*, *timeless*, *cryptochrome*, *clock* and *bmal1*) that act in positive and negative feedback loops to generate molecular rhythms that are imposed on the rest of the body by neural and humoral connections.^1^ The detailed description of the molecular generation of circadian rhythms can be found elsewhere.^1^ However, it is important to note two salient points. First, the genes involved and the way in which they interact to drive rhythmicity are extremely highly conserved across taxa. Thus, an understanding of how the clock works and how it is affected by external factors such as light and drugs in invertebrate models (e.g., the fruit fly) holds direct relevance for understanding these effects in humans. Second, there are clocks throughout the body, not just in the SCN.^1^ In fact, we have clocks in all our peripheral tissues, including the liver, heart and gut. These peripheral clocks are normally kept in time by the central SCN-based oscillator, and the synchrony of the peripheral clock systems with the central oscillator is as important as the rhythmicity of the central clock for our health and wellbeing.

Although the circadian clock in the SCN drives rhythmicity in all mammals, it is not insensitive to environmental influences. Our endogenous circadian clocks tick with an inherently inaccurate period relative to the length of a day (in humans it is 24.3 h) but are adjusted on a daily basis primarily by environmental light, which shifts the clock in the morning and evening. Light, detected by the combination of rod and cone photoreceptors and by the circadian photopigment melanopsin, is the primary entraining mechanism that shifts our clocks, and those of other organisms, to 24 h on a daily basis.^11^ In addition to light and anaesthetic drugs, other factors, including food and exercise, affect the clock.^3^

There are numerous outputs from the SCN-based clock, the best characterised of which is a multisynaptic pathway to the pineal gland, which produces melatonin under the direct control of the SCN. Circulating endogenous melatonin concentrations peak at night (at approximately 100 pg ml^–1^) and are directly suppressed by night-time light exposure. Endogenous melatonin feeds back onto melatonin receptors in the SCN, reinforcing daily rhythms. Pharmacological doses of exogenous melatonin can shift our circadian clocks by acting on these same receptors in the SCN.^12^

The circadian system is perhaps best thought of as a central clock in the brain, sitting neatly above the optic chiasm, ticking away with an inherently non-24-h period but receiving light from the eye to adjust its period each day, and then exerting its influence on clocks in the peripheries, including the cardiovascular, gastrointestinal and excretory systems. It is perhaps unsurprising then that the clock can have significant effects on the absorption, distribution, metabolism and excretion of drugs used in anaesthesia and thus influence their efficacy and duration of action.

## The effect of the circadian clock on anaesthetic drug action

The pharmacokinetics and pharmacodynamics of many anaesthetic, analgesic, and neuromuscular blocking drugs show marked circadian variation (reviewed in Poulsen and colleagues^3^). In practice, this can translate into differences in dosing regimens, but also changes in the duration of recovery times in the post-anaesthesia care unit.^13^

Rhythmic shifts in gastric acidity, motility and splanchnic blood flow influence drug absorption, whereas daily changes in membrane permeability and plasma protein concentration modify drug distribution. Likewise, hepatic blood flow and enzymatic activity have strong time-of-day oscillations, contributing to substantial variation in metabolic clearance. Renal elimination is similarly affected by rhythmic changes in perfusion.^14^ These fluctuations in pharmacokinetics are complemented by circadian modulation of pharmacodynamics. Receptor density and function, including β- and α_1_-adrenoceptors, μ-opioid receptors and 5-HT_1B_ autoreceptors, vary across the 24-h cycle, altering tissue responsiveness to commonly used drugs.^15^

Although time-of-day effects have been characterised for all of the classes of drugs used in anaesthesia (reviewed in Poulsen and colleagues^3^), the most consistent evidence relates to local anaesthetic drugs. The duration of action of local anaesthetic drugs is greatest in mid-afternoon, around 15:00, across multiple drugs and routes of delivery. These consistent patterns are likely to reflect combined circadian influences on membrane permeability, potassium flux, and hepatic clearance: studies show peak local anaesthetic plasma concentrations and trough potassium efflux occurring in this same time window.^2^^,^^16^^,^^17^ Notably, a recent clinical study from Greece involving 90 women undergoing spinal anaesthesia with a single shot intrathecal levobupivacaine and fentanyl for caesarean recruited patients over an entire 24-h period.^18^ Data were analysed in three groups (08:00 to 16:00, 16:00 to midnight and midnight to 08:00), and the shortest duration of analgesia was found to occur between midnight and 08:00, whereas the longest time to first request for analgesics occurred between 08:00 and 16:00.^18^

Given the clear quantitative endpoints associated with neuromuscular block, some of the most compelling data showing circadian effects on drugs used in anaesthesia come from the study of neuromuscular blocking drugs. Daily variation in the action of pancuronium was first described in patients undergoing cholecystectomy who were reported to have a greater requirement for pancuronium at 09:00 than at other times of the day (reviewed in Cheeseman and colleagues^5^). These findings were extended by our group in 2007 in work showing that an intubating dose of rocuronium resulted in a blockade lasting 30% longer in the morning (between 08:00 and 11:00) than in the afternoon (between 14:00 and 17:00).^5^ Although the mechanisms of this effect are unclear, likely causes include rhythmic variations in hepatic metabolism, neuromuscular transmission and receptor availability.

## The effects of the circadian clock on pain

In addition to the effect of the circadian clock on anaesthetic drug action, it is important to highlight the circadian rhythms that exist in pain responses. Initial studies showed that morphine-induced analgesia in mice was maximal during the active phase and that this was partly caused by an underlying rhythm in pain perception. These studies were followed up by a plethora of laboratory studies, including data showing that the interaction of morphine, naloxone and N^G^-nitro-L-arginine methyl ester on pain sensitivity changes depending on the circadian rhythm of pain (with a 60% change in analgesia).^2^^,^^14^ In a recent systematic review including 39 studies of circadian patterns in human pain, the authors found evidence for clear circadian patterns of pain in nociceptive, neuropathic, central and mixed pain states.^19^ Patients experiencing postoperative pain, fibromyalgia, trigeminal neuralgia and migraines primarily report higher pain scores in the morning, whereas those experiencing temporomandibular joint pain, neuropathic pain, labour pain, biliary colic and cluster headaches show higher pain scores in the evening or at night.^19^

However, some of these daily variations in pain are complicated. For example, in a recent study of chronic pain, all patients showed variation in pain across the day but with different patterns. Although most patients experienced more pain on waking, one-third of patients with chronic pain had the highest pain scores in the late afternoon and early evening.^20^

## The effect of anaesthesia (and surgery) on the circadian clock

Patients frequently experience pronounced fatigue and disrupted sleep after surgery.^21^ Although multiple perioperative factors, including noise, pain and anxiety, contribute to altered postoperative sleep, one driver that is increasingly recognised as being important is anaesthesia-induced disruption of the circadian clock.^3^^,^^22^^,^^23^ Circadian disruption and its associated sleep disturbance are linked with adverse effects on mood, cognitive performance, and pain and increased inflammation and impaired immune function.^7^^,^^24^ Thus, reducing clock disruption is a potentially important way to improve postoperative patient wellbeing and expedite recovery.

### The effect of anaesthesia on circadian rhythms in animal behaviour

Hospitalisation, surgery, and disease are all known to influence circadian rhythms (e.g., the lack of cortisol rhythms in patients in ICU).^25^ It is generally considered unethical to give anaesthesia to humans unless medically required, but animal models have provided a powerful insight into the effects of anaesthesia on the circadian clock. At least 20 behavioural studies in vertebrates and invertebrates have shown that anaesthetic agents interact with and disturb the circadian timing system.^3^

However, considerable variation can be seen between studies in terms of the magnitude and direction of the clock shifts. This is likely to result from the use of a range of different protocols and different anaesthetic agents (e.g. gamma-aminobutyric acid [GABA] agonists, such as propofol, inhalational anaesthetic agents and ketamine). In spite of the differences across studies, some common effects are seen.

#### Anaesthesia during the active vs rest period

Although most studies demonstrate a shifting effect of anaesthesia during the active phase (day for diurnal animals and night for nocturnal animals), less than half show an effect during the rest phase (and many of these are smaller in magnitude than the shifts during the active phase). These time-of-day effects can also be observed in other aspects of anaesthetic potency, including emergence from anaesthesia. A recent study examining the loss and recovery of the righting reflex of mice after the administration of isoflurane and sevoflurane showed a faster recovery (emergence) in mice anaesthetised during their active phase (night) than their inactive phase (day).^26^

#### The effect of anaesthesia in different environmental conditions

Most studies show a delay of the circadian clock in time-isolation conditions (the absence of daily light cycles), whereas few show a delay in the presence of strong natural light cycles. This delay may reflect the opposing effects of light and anaesthesia on the circadian clock, particularly in the morning. In diurnal animals, such as the honey bee, large (3-h) delays in the circadian clock after isoflurane anaesthesia can be abolished by the concomitant administration of strong light (10,000 lux).^4^ Findings such as these may have implications for the prevention of anaesthesia-induced clock shifts in patients by the use of intraoperative light exposure (which can be effective even through closed and taped eyes).

#### General anaesthesia may inhibit light-entrainment of the circadian clock

There is evidence that the interaction between light and anaesthesia can be bidirectional and that in certain situations anaesthesia blocks the shifting effect of light on the circadian clock. This is particularly true of nocturnal models, such as mice and rats. For example, the administration of light elicits strong phase shifts in the mouse circadian clock, with a 4-h light pulse shifting the clock by up to 3 h.^27^ However, when isoflurane is given together with light, the shifting effect of light is reduced by approximately 50%.^27^ The pathway via which light shifts the mammalian circadian clock (the retinohypothalamic tract) relies heavily on N-methyl-D-aspartate (NMDA) receptor activation. Furthermore, GABA receptors are involved in both clock coupling (in an inhibitory *and* excitatory manner) and shifting the clock at a molecular level. Thus, drugs that act on NMDA and GABA can block the pathway of light to the clock.^28^

### The effect of anaesthesia on circadian rhythms in clock gene expression

Fifty-six of the top 100 best-selling drugs in the US (including all of the top seven: aripiprazole, esomeprazole, duloxetine, rosuvastatin, seretide, adalimumab and etanercept) directly target the protein product of a circadian clock gene.^29^ The effects of anaesthesia on behavioural rhythms in both vertebrates and invertebrates can be explained directly by studies showing that these agents act on the expression patterns of the clock genes. Inhalational and i.v. anaesthetic agents have been shown to induce shifts in the expression patterns of clock genes in vertebrates and invertebrates.^23^^,^^30^ These effects occur in both central and peripheral clock tissues.^30^^,^^31^

An understanding of the mechanistic basis of these effects has emerged recently from studies on sevoflurane and ketamine. Sevoflurane has been shown to directly reduce the acetylation of regulatory regions (known as E-boxes) of the key clock gene *period*, reducing its binding to its protein partner *clock* and thus affecting the phase of the molecular loop that drives behaviour.^32^ Ketamine modulates the ability of the clock protein dimer CLOCK-BMAL1 to activate transcription of other clock genes in a dose-dependent manner, again changing the period of the fundamental biochemical loop that drives rhythms in physiology and behaviour.^33^

## The effect of anaesthesia on patients’ circadian clock and postoperative recovery

How anaesthetics affect the circadian clock in animal models and how these effects are mediated provide an insight into potential treatment approaches that cannot always be gained through clinical studies. However, the ultimate goal is, of course, to determine whether patients experience clinically important levels of clock-driven sleep disruption and how this may be mitigated.

### The hospital environment

Gaining a clear understanding of the effects of anaesthesia (and surgery) on the circadian clock has been difficult owing to the many factors that affect the clock in the hospital setting. Underlying disease states, anxiety, and pain all result in sleep and circadian disruption. Therefore, understanding the effect of anaesthesia (and surgery) on patients with pre-existing clock disruption is difficult. Furthermore, the hospital environment is highly disruptive and often lacks the strong ‘clock-entraining’ light cycles that are experienced in everyday life. Even nursing care can result in circadian disruption, when patients are woken through the night to have observations taken or asked their pain scores. Different research groups have taken different approaches to investigating the effects of anaesthesia on the clock. For example, our group has studied circadian disruption in patients undergoing donor nephrectomy, who represent patients undergoing major surgery that is not medically required and in whom baseline sleep and circadian rhythms are undisturbed. Through efforts such as these, there is emerging evidence showing that the circadian clocks of patients are disrupted by anaesthesia and that these disruptions are associated with longer durations of recovery. Thus, treating clock disruption may represent an effective way of expediting postoperative recovery.

### Effects on sleep

Initial studies provided evidence that sleep-wake rhythms and rapid eye movement sleep cycles were disrupted in patients after general anaesthesia for laparoscopic cholecystectomy and major abdominal surgery.^34^ These findings were bolstered more recently by studies showing that sleep timing, sleep quality and sleep architecture are all affected by general anaesthesia for a range of procedures, including orthopaedic, plastic and dental operations.^35^

### The persistence of sleep disruption

More equivocal evidence is also emerging that perioperative sleep disorders can persist after discharge from hospital. For example, data from a 2024 study found that 11% of surgical patients, but only 5% of patients undergoing regional anaesthesia, have sleep disruption that lasts several weeks and that subjectively measured sleep timing does not normalise until at least 1 week after surgery.^36^^,^^37^ However, other studies, particularly in children, have failed to identify persistent sleep disruption.^38^ Further studies are required, but the notion that circadian disruption not only occurs but also fails to resolve quickly after discharge highlights the potential importance of interventions to improve postoperative sleep.

### The disruption of other physiological and humoral measures

The effects of anaesthesia on the clock do not seem to be restricted to effects on the sleep-wake cycle. Studies on daily rhythms in the heart rates of patients have shown clear postoperative disruptions after both bariatric and cancer surgery, which persist for several days and are exacerbated (i.e. higher peaks and troughs) in patients with cancer who experience postoperative deterioration compared with those without deterioration.^39^

As a key marker of the circadian clock, endogenous melatonin production can be monitored in plasma, saliva, or urine to quantify circadian disruption. Acute suppression of melatonin production by anaesthesia is not surprising as many drugs directly inhibit enzymes such as *N*-acetyltransferase that are key to its synthesis. However, it is the chronic effects of anaesthesia on the daily rhythm in melatonin production that provide evidence for an effect on the clock. Patients undergoing general anaesthesia for hip replacement surgery have been shown to have a larger disruption to their melatonin rhythm postoperatively than those having regional anaesthesia.^40^ The disruption in melatonin production takes several days to return to preoperative levels.^40^

### The potential efficacy of light and melatonin as treatments

Light is unequivocally the most powerful synchroniser of our circadian clocks, and as such it can be used in a wide range of settings to improve circadian ‘entrainment’. The postoperative setting is no exception to this. Strong morning light (e.g. >10,000 lux of white light) for at least 30 min is highly effective in the entrainment of patients’ circadian rhythms.

The suppression of endogenous melatonin production and the disruption of the clock has been associated with an increase in postoperative delirium.^41^ However, evidence for the positive effects of pharmacological doses of melatonin is limited, and the mechanisms behind the claimed positive effects on postoperative patient wellbeing remain unclear. The administration of exogenous melatonin may reduce delirium.^42^ Furthermore, studies have shown melatonin can reduce pain and anxiety.^42^ A Cochrane review in 2020 found that melatonin is as effective as benzodiazepines in reducing preoperative anxiety in adults measured 50–120 min after dosing.^42^ Although the risks associated with giving melatonin are very low, we would advocate further studies before the widespread use of melatonin as an anxiolytic or analgesic is promoted. However, if these findings are demonstrated to be robust, pre- and postoperative use of melatonin could form part of a suite of approaches to improve postoperative wellbeing. If effective, the mechanism would likely be by melatonin’s action in reinforcing circadian rhythms and potentially as a mild initiator of sleep rather than any inherent analgesic or anxiolytic property.

### Other factors to improve postoperative rhythms

Given its efficacy in reducing postoperative nausea and vomiting, postoperative pain, and opioid requirements, the use of dexamethasone in anaesthesia has increased in popularity. However, interestingly, as a potent glucocorticoid analogue, dexamethasone not only suppresses melatonin (by affecting *N-*acetyl transferase) but has also been shown to shift the timing of expression of clock genes and in particular peripheral clocks in the liver and kidney, which can desynchronise them from the central SCN-based clock.^43^

As noted earlier, food and exercise also shift the clock. These effects are mediated by peripheral clocks in the liver, gut and adipose tissue and, as such, are influenced by drugs such as dexamethasone that act on peripheral clock systems.^43^ These observations are also relevant for the perioperative period, especially in critically ill patients. Preoperative fasting frequently extends for periods beyond 12 h, especially in patients awaiting acute surgery. Given the powerful role of food-timing in synchronising peripheral clocks, prolonged preoperative fasting may contribute to internal desynchrony between central and peripheral oscillators. When compounded by the suppression of physical activity inherent in hospitalisation and the disruption to the light-dark cycle commonly seen in hospital environments, the perioperative patient effectively loses three major clock-entraining signals (light, food and exercise) simultaneously.

One final comment is around the usefulness of α_2_-agonists and dexmedetomidine to support circadian rhythmicity and reduce delirium.^44^ Dexmedetomidine seems to act specifically on endogenous sleep-promoting pathways to exert its effect and, as such, causes less disruption than many other anaesthesia adjuncts.^44^ This, coupled with very recent evidence (albeit in mice) that dexmedetomidine can accelerate the daily adjustment of the clock to the environmental light cycle, suggests that its use might provide a further pharmacological means to support circadian rhythmicity and postoperative patient health.^45^

## Conclusions

The field of circadian biology has practical implications that are emerging as relevant to anaesthetists for clinical practice. The first is that the time of the day can explain a substantial portion of the interpatient variability in response to drugs used in anaesthesia. This difference cannot be solely attributable to natural states of sleepiness, as the effects are evident in all classes of anaesthesia drugs, including neuromuscular blocking drugs. There is consistent evidence showing the duration of analgesia provided by local and regional anaesthetics in particular is longer in the afternoon than at other times of the day. Importantly, patients experiencing postoperative pain report higher pain scores in the morning, whereas those experiencing neuropathic and labour pain show higher scores in the evening or at night.

Despite conflicting data on different agents, there is consistent evidence that general anaesthesia disrupts the circadian clock, the rhythms it controls, and its daily adjustment to the light cycle. Regional anaesthesia seems less disruptive in this regard. Furthermore, the disruption to the clock is associated with both a decrease in patients’ wellbeing and an increase in the incidence of postoperative delirium.

Although clinicians may have little (if any) control over the time of the day at which operations occur or the disruptions that result from anaesthesia and surgery, there is an opportunity to try and maximise environmental factors known to help keep the circadian clock well adjusted. These include strengthening light-dark cycles, reducing prolonged fasting times, and minimising overnight disruptions for patients.

In terms of pharmacological approaches, regional techniques seem beneficial, and giving melatonin and α_2_-agonists, such as dexmedetomidine, may be considered.

## MCQs

The associated MCQs (to support CME/CPD activity) will be accessible at www.bjaed.org/cme/home by subscribers to *BJA Education*.

## Declaration of generative AI and AI-assisted technologies in the writing process

During the preparation of this work, the authors used Notebook LM to draw the infographic. After using this tool/service, the authors reviewed and edited the content as needed and take full responsibility for the content of the published article.

## Declaration of interests

The authors declare that they have no conflicts of interest.
